# Associations Between Sleep State Misperception and Emotional States in Middle‐Aged Adults: A Community‐Based Cross‐Sectional Study

**DOI:** 10.1155/bn/6132707

**Published:** 2026-09-24

**Authors:** Xiaoyu Sun, Hideki Miyaguchi, Chinami Ishizuki, Hitoshi Okamura

**Affiliations:** ^1^ Graduate School of Biomedical and Health Sciences, Hiroshima University, Hiroshima, Japan, hiroshima-u.ac.jp; ^2^ Department of Rehabilitation, University of Kochi Health Sciences, Kochi, Japan

**Keywords:** actigraphy smartwatch, emotional states, health prevention, health promotion, middle-aged, sleep misperception

## Abstract

Several studies have explored sleep discrepancy as a key to understanding insomnia, linking it to attentional bias, arousal, distorted processing, depression, and anxiety. However, further research is needed to understand whether discrepancies in sleep perceptions observed in a community‐dwelling middle‐aged population, vulnerable to stress, were related to mood and other social factors. We used self‐report questionnaires and smart watches to investigate the discrepancies between perception of sleep and objective data among 32 community‐dwelling middle‐aged individuals. We analyzed the relationship between sleep discrepancies and psychosocial factors and compared emotional state scores across different sleep tendencies to examine whether sleep discrepancy could serve as a potential indicator for identifying variations in psychological well‐being. Emotional states associated with sleep discrepancy perception among middle‐aged adults included tension–anxiety (TA) (*r* = −0.361), depression–dejection (*r* = −0.475), and total mood disturbance (TMD) (*r* = −0.287). In the regression model, sex, TMD, and self‐rated health were significant predictors of perceived sleep discrepancy (adjusted *R* ^2^ = 0.392, *F* = 7.873, *p* = 0.001). Group comparisons showed that those with discrepancies between subjective and objective sleep measures had higher TA and Fatigue–Inertia levels compared with those with concordant subjective and objective sleep, and that age influenced these differences. These findings underscore the association between emotional states and sleep misperception. Sex, self‐rated health, and TMD scores were independent predictors of sleep misperception index in middle‐aged adults. Future longitudinal studies should clarify the causal relationship between the two and expand the possibilities of using actigraphy devices as tools for sleep monitoring and health prevention in daily settings.

## 1. Introduction

Sleep state misperception refers to the discrepancy between subjective and objective assessments of sleep, encompassing the magnitude and direction of this divergence. It is typically quantified using indices derived from differences in sleep parameters such as total sleep time (TST), sleep onset latency, and wakefulness after sleep onset (WASO) [1, 2]. Previously, this phenomenon was described as paradoxical insomnia, which the *International Classification of Sleep Disorders, Third Edition* [3], defines as a complaint of severe sleep disturbance despite the absence of objective evidence supporting the reported severity. Accordingly, early research primarily focused on clinical populations with insomnia or affective disorders, whereas community‐dwelling individuals without clinically diagnosed insomnia or psychiatric disorders were rarely included. Consequently, current understanding of sleep state misperception has largely been derived from clinical samples, which may limit the generalizability of previous findings and potentially skew conclusions regarding the prevalence and nature of this phenomenon in the general population. However, accumulating evidence suggests that sleep state misperception also occurs in nonclinical populations, where it may exist as a state ranging from sleep underestimation to overestimation, rather than as a stable pathological trait, reflecting individual differences in the cognitive and emotional processing of sleep‐related information [4, 5]. Since sleep state misperception is closely associated with psychological factors, elucidating its direction and magnitude in relation to different emotional states may deepen our understanding of the cognitive and emotional mechanisms underlying sleep perception and provide a theoretical basis for the recognition and management of emotional problems in community settings.

At the population level, midlife has been recognized as a vulnerable period for mental health and well‐being. Compared with younger adults, individuals in midlife tend to exhibit a relative decline in mental health indicators [6–8], face a higher risk of suicide mortality, and respond relatively less favorably to psychotherapy [9, 10]. At the same time, midlife represents a critical life stage during which protective and harmful factors for mental health accumulate, and the mental health foundations established during this period may have long‐term implications for well‐being in later life [11]. From a public mental health perspective, identifying and managing mental health problems in middle‐aged populations is therefore particularly important. However, timely identification of psychological distress in community settings remains challenging. Stress is inherently subjective, and prolonged exposure to high‐demand environments may lead to sustained activation of the stress response system, to which the brain gradually adapts, attenuating individuals′ subjective awareness of their own stress states [12, 13]. Furthermore, chronic stress may impair prefrontal regulatory functioning, weakening self‐monitoring, and emotional regulation capacities, and thereby hindering accurate recognition of one′s psychological state [14]. Psychological stress may also manifest as somatic symptoms, further complicating its recognition in the absence of specialized assessment tools [15]. Given these challenges, emotional states may serve as more accessible indicators of underlying stress‐related psychological changes. Exposure to stress influences emotional expression through hormonal regulation, manifesting as increased negative affect, heightened emotional reactivity, and reduced emotion regulation capacity. In community settings where professional assessment tools are often unavailable, identifying observable and measurable indicators that reflect emotional states may facilitate early detection of psychological changes and support the management of stress‐related conditions in middle‐aged populations.

A substantial body of epidemiological research has demonstrated that sleep parameters are closely associated with physical and psychological health outcomes [16–19]. Beyond objective sleep characteristics, the discrepancy between subjective and objective sleep measures has increasingly been recognized as an important indicator of sleep‐related cognitive and emotional processes. The sleep state misperception index, one of the most commonly used metrics to quantify this discrepancy, is considered a clinically meaningful indicator that may reflect an individual′s underlying psychological state [2]. Such discrepancies may arise from cognitive and emotional processes rather than being determined solely by objective sleep characteristics. Age‐related changes in cognitive function, including declines in memory consolidation and time perception, may affect how individuals estimate their sleep duration. Anxiety‐related attentional biases, negative interpretations of nocturnal awakenings, and heightened self‐monitoring may further distort subjective sleep evaluation, whereas physiological stress responses including hyperarousal and alterations in sleep architecture may widen the gap between perceived and measured sleep [20]. For middle‐aged individuals frequently exposed to occupational, familial, and social stressors, these processes may be particularly pronounced, as sustained stress activation can amplify emotional reactivity and reinforce maladaptive cognitive appraisals of sleep [21]. Critically, sleep discrepancy may not only reflect but also perpetuate emotional dysregulation; persistent misperception of sleep adequacy can sustain negative affective states, heighten arousal, and impair emotional regulation capacity through continued activation of stress‐response systems [22]. In community‐dwelling middle‐aged adults, such subclinical emotional changes may be difficult to detect through conventional assessments, rendering sleep misperception index a potentially sensitive and accessible marker of early psychological strain. Nevertheless, the specific associations between sleep state misperception and distinct emotional states in this population remain inadequately understood, and whether the direction and magnitude of sleep discrepancy are differentially associated with distinct emotional profiles remain unclear. The present study, therefore, is aimed at examining these associations in a sample of community‐dwelling middle‐aged adults, with the goal of informing early psychological health screening, supporting community‐based intervention strategies, and providing a theoretical foundation for future clinical and cognitive neuroscience research.

Building on this framework, converging evidence suggests that different emotional states may influence sleep state misperception through distinct mechanisms. High‐arousal negative emotional states, including tension, anxiety, and anger, heighten physiological activation through increased sympathetic nervous system activity and hypothalamic–pituitary–adrenal axis dysregulation, elevating cognitive and somatic arousal at sleep onset and selectively amplifying the subjective experience of wakefulness, thereby biasing estimates of sleep duration downward [23]. In contrast, depressive affect, characterized by low arousal and negative valence, may influence sleep perception through a distinct cognitive pathway; negatively valenced memory encoding of nocturnal experiences and ruminative processing are associated with selective recall of wakefulness and underestimation of actual sleep time. Evidence from community and subclinical samples supports the association between depressive affect and subjective underestimation of sleep, even after controlling for objective sleep parameters [4]. Low‐arousal states, including fatigue and lethargy, may instead reduce interoceptive sensitivity, attenuating awareness of brief nocturnal awakenings and promoting positively biased sleep evaluations [24–26]. The predicted direction of association for vigor‐based states was treated as exploratory, given that its high‐arousal and positive‐valence profile may yield competing effects on sleep perception. Based on this evidence, we hypothesized that emotional states associated with high arousal and negative valence, including tension, anxiety, and anger, as well as low‐arousal depressive affect, would each be associated with underestimation of sleep duration through their respective mechanisms, whereas fatigue‐related states would be associated with overestimation. Furthermore, higher levels of tension, anxiety, and depression were expected to be associated with larger absolute sleep discrepancies, whereas higher vigor was expected to be associated with smaller discrepancies, consistent with its established association with accurate interoceptive awareness.

Over the past decade, the rapid development and widespread adoption of consumer sleep technologies (CSTs), together with the establishment of standardized testing frameworks, have made reliable long‐term sleep monitoring in community settings increasingly feasible, thereby enhancing the potential for improved population‐level sleep health assessment [27]. Although polysomnography (PSG) remains the gold standard for sleep assessment [28], its laboratory‐based nature renders it impractical for continuous monitoring in real‐world community contexts [29]. Conventional actigraphy devices typically rely on movement‐based algorithms, such as the Cole–Kripke or Sadeh methods, and limited sensor configurations. In contrast, high‐quality CSTs employ multisensor architectures integrating accelerometry, photoplethysmography, and skin temperature sensors, alongside algorithms trained on substantially larger datasets, enabling more comprehensive sleep characterization [30]. To facilitate long‐term sleep monitoring in community settings and to explore scalable approaches for health assessment outside clinical settings, we selected the Polar Vantage v2 smartwatch as the measurement instrument. Independent validation studies have demonstrated acceptable performance of the Polar Vantage series for TST and WASO against PSG‐grade references, with sleep–wake detection accuracy and agreement metrics comparable to other widely used CSTs such as the Oura Ring [31–33]. Nevertheless, like other CSTs, the Polar Vantage v2 has limitations in sleep stage classification and sleep onset latency estimation, and direct PSG validation of this specific model in adult community samples remains limited. Despite these constraints, CSTs represent a pragmatic and increasingly validated approach for capturing habitual sleep patterns in healthy populations when PSG is not feasible in long‐term real‐world settings [31–33]. Our study sought to extend the application of wrist‐worn CSTs in healthy community populations and to provide a basis for developing standardized approaches to sleep and psychological state monitoring outside clinical settings.

Therefore, this study is aimed at clarifying the relationship between sleep state misperception index and emotional states in community‐dwelling middle‐aged individuals. Our study aims to establish an empirical link between emotional states and quantifiable indicators by examining the extent to which specific metrics reflect meaningful variations in emotional functioning.

## 2. Materials and Methods

### 2.1. Participants

Participants were recruited through Hiroshima City′s public relations channels and online platforms. The age range of 45 to 64 years was determined in accordance with Japan′s national health promotion strategy [34], which categorizes this life stage as middle age based on social roles and the demographic structure of an ageing society. This study was approved by the Ethical Committee for Epidemiology of Hiroshima University (approval No: E2024‐0193), and written informed consent was obtained from all participants prior to enrollment. Participation was entirely voluntary and no financial compensation was provided.

Participants were included if they met all of the following criteria: 1) age between 45 and 64 years; 2) noneligibility for long‐term care insurance services; 3) self‐reported subjective dissatisfaction with sleep, with no prior clinical diagnosis of sleep‐related breathing disorders, and a history of clinical PSG performed at a sleep clinic or hospital without a diagnosis of obstructive sleep apnea (OSA) or other sleep‐related breathing disorders recorded at that time; 4) reported stable sleep habits, absence of typical OSA‐related symptoms, stable body weight, and no development of high‐risk conditions such as secondary hypertension; 5) habitual sleep duration of at least 5 h per night between 8 p.m. and 9 a.m.; 6) body mass index (BMI) less than 35 kg/m [2]; 7) no excessive daytime sleepiness; 8) no caffeine or alcohol consumption within 6 h prior to bedtime throughout the assessment period; and 9) nonsmoker status. Participants were excluded if they met any of the following criteria: 1) a diagnosis of psychiatric disorders; 2) suspected difficulty with verbal communication or cognitive impairment, as judged by the researchers based on conversational assessment; 3) a serious illness diagnosed or experienced within the past 3 months, including dementia, neurodegenerative disease, Parkinson′s disease, fibromyalgia, major vascular disease, arrhythmia, or cerebrovascular disease; 4) inability to complete study‐related measurements, such as difficulty sleeping while wearing the monitoring device; 5) current use of hypnotics or sleep aids, or a history of continuous positive airway pressure therapy or oral appliance therapy for sleep‐related breathing disorders; and 6) premenopausal or perimenopausal status in women.

### 2.2. Questionnaires

#### 2.2.1. Basic Information

Basic demographic and health information, including age, sex, occupation, height, weight, frequency of daily exercise, educational background, marital status, cohabitation status, and past medical history, including chronic conditions and menstrual status in women, was collected using a general information questionnaire.

#### 2.2.2. Profile of Mood States, Second Edition‐Brief (POMS 2.0)

The POMS 2.0 was used for assessing emotional states and stress‐related mood fluctuations, and the standardization, reliability, and validity in the Japanese population has been confirmed [35]. The POMS 2.0 is a 35‐item self‐reported questionnaire rated on a 5‐point Likert scale. The scale assesses seven mood subscales—tension–anxiety (ta), anger–hostility (ah), depression–dejection (dd), fatigue–inertia (fi), confusion–bewilderment (cb), vigor–activity (va), and friendliness (f). Each mood dimension is converted into a standardized T‐score, and the total mood disturbance (TMD) score is computed by summing the negative mood subscales and subtracting the VA score. The TMD score summarizes overall mood state, with higher values indicating greater emotional distress.

#### 2.2.3. Insomnia Severity Index (ISI)

The ISI is a 7‐item self‐reported scale capable of assessing individuals′ perception of sleep quality, including sleep onset, maintenance, early awakening, daily functioning, and perceived sleep‐related distress. Previous research has shown that, beyond the fields of sleep medicine and insomnia, it has also been applied in studies examining subjective sleep quality and its relationship with the patient′s current condition [36]. The scores range from 0 to 28, with higher scores indicating greater distress. Existing reports have indicated that individuals with a score of 8 or higher are poor sleepers [37].

#### 2.2.4. Sleep Log

Participants provide a daily sleep log according to the 9‐item consensus sleep log (excluding the 9th comment item), reporting bedtime, wake‐up time, TST, sleep onset latency, WASO, and subjective restorative sleep [38]. Participants completed a morning sleep log within 10 min after a morning synchronization between the Polar watch and a smartphone‐based app.

#### 2.2.5. Self‐Rated Health (SRH)

SRH was used to evaluate participants′ perceived health status by answering the survey question “What is your current state of health?”. We collected responses using a 5‐point Likert scale ranging from “very poor” to “very good.” Since less than one respondent chose “very poor,” we combined the “very poor” and “poor” categories for analysis. We grouped “good” and “very good” responses under the positive SRH group and “not so good” and “poor” responses under the negative SRH group.

### 2.3. CSTs

Daily sleep duration was measured using the prototype Polar Vantage v2 sportwatch (Polar Electro Oy, Finland) equipped with the validated Sleep Plus Stages algorithm, which integrates accelerometer and optical heart rate sensor data. This device features a triaxial accelerometer (sampling rate of 52 Hz) and a proprietary photoplethysmography sensor to detect pulse intervals. Data were uploaded to a web‐based service using a Polar proprietary algorithm to identify sleep periods and classify sleep stages in 30‐s epochs, which met the criteria and complied with the American Academy of Sleep Medicine′s standards for the analysis of different sleep stages. The measured sleep variables included sleep onset and offset times, sleep onset latency, TST, and WASO.

### 2.4. Sleep State Misperception

The degree of sleep state misperception was mainly evaluated using the TST misperception index, defined as (subjective TST − objective TST) divided by objective TST [18]. Participants were classified into overestimation or underestimation groups based on the positive or negative mean misperception indices, respectively.

### 2.5. Procedure

After completing the baseline questionnaire, participants were provided with a Polar Vantage V3 (Polar Electro Oy; Kempele, Finland). Before data collection, participants received instructions and device training to ensure proper use. Participants were instructed to maintain, as much as possible, a consistent sleep schedule in a dark, quiet, and comfortable environment. The first 3 days served as an adaptation period to familiarize participants with the device and reduce immobile wakefulness related to discomfort or lying awake, with formal sleep monitoring beginning on the fourth day. Participants were instructed to wear the smartwatch continuously, except during bathing or smartwatch charging. At night, they went to bed according to their self‐determined sleep schedule. In the morning, if they were not already awakened at their requested wake‐up time, they were awakened by an alarm. Upon waking, participants synchronized the device data with the mobile application and completed a standardized sleep log within 10 min.

To minimize the impact of low sensitivity on device‐based sleep–wake detection, participants were instructed to avoid reading books, watching videos, responding to messages, and chatting and instead remain seated quietly with minimal movement during the 30‐min period prior to bedtime and maintain consistent sleep routines. Participants also installed a smartphone application (app usage) that recorded screen‐on time and user interactions. In the event of nocturnal awakenings, participants were asked to unlock their smartphones to mark periods of immobile wakefulness. Daily data were subsequently cross‐checked against screen‐use logs to adjust for potential wakefulness.

**Figure 1:**
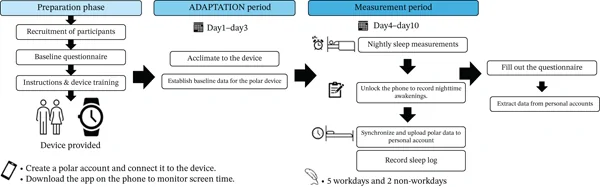
Research procedure.

Sleep was monitored for 7 consecutive days, commencing on Sunday night and concluding the following Sunday morning. Participants labeled each night as a workday or nonworkday. The monitoring period included two nonworkday nights and five workday nights. Depending on the nature of their occupations, participants recorded work nights either consecutively or nonconsecutively (Figure 1).

### 2.6. Statistical Analyses

An a priori power analysis was conducted using G ^∗^Power Version 3.1.9.6 to estimate the required sample size. Assuming a significance level of *α* = 0.05 and a statistical power of 0.80, the estimated minimum sample size required for the analysis was 26 participants.

Previous research indicates that measurement for at least four nights, consecutive or nonconsecutive, provides a reliable estimate (ICC > 0.8) of mean TST and its variability. However, obtaining a reliable estimate of average sleep time on nonworkdays also requires at least 4–5 days of data. Therefore, nonworkdays were not included in the present analysis, and only workday sleep data were analyzed. Sleep parameters were calculated as the mean values across valid recording nights for each participant to account for day‐to‐day variability in sleep patterns. Potential outliers were identified using the interquartile range (IQR) method, with values falling below Q_1_ − 1.5 × IQR or above Q_3_ + 1.5 × IQR considered outliers and excluded. Participants with fewer than four valid nights after outlier removal were excluded from further analyses. Accordingly, the mean TST misperception index across the five workday nights was calculated as the primary outcome measure. Participants were classified into overestimation and underestimation groups based on the positive or negative values of their TST sleep misperception index, respectively.

The normality of continuous variables was assessed using the Shapiro–Wilk test. All normally distributed variables are expressed as means and standard deviations (M ± SD), whereas nonnormally distributed variables are presented as medians. Independent‐samples *t*‐tests were used to compare variables between groups when the assumption of normality was met, whereas Mann–Whitney *U* test was applied for nonnormally distributed variables. Fisher′s exact test was used to examine differences in categorical variables. Because the sleep state misperception index and several emotional variables did not satisfy the assumption of normality, spearman correlation analyses were conducted to examine the relationships between mood dimensions, age, ISI scores, and the misperception index. Potentially relevant factors from three domains—sociodemographic and health‐related variables (age, sex, occupation, height, weight, frequency of daily exercise, educational background, and marital status), mood dimensions assessed using the POMS (TA, AH, CB, DD, FI, VA, and TMD score), and SRH status were entered into a multiple regression analysis to determine variables independently associated with the misperception index. Independent variables were selected based on prior studies and clinical considerations, and all variables were entered simultaneously using the forced‐entry method. Model adequacy was assessed based on probability values, adjusted *R* ^2^, and inspection of residuals for normality.

In addition, participants were classified into three groups based on subjective and objective sleep assessments. Subjective sleep status was classified as negative or positive based on whether their ISI score was ≥ 8. Objective sleep status was considered negative if sleep onset latency exceeded 30 min on more than three nights during the monitoring week or if participants experienced three or more awakenings after sleep onset, each lasting > 30 min; otherwise, the objective sleep status was considered positive. Participants whose subjective and objective sleep statuses were positive were included in the favorable sleep group, those with mismatch between subjective and objective sleep status (i.e., positive subjective but negative objective and negative subjective but positive objective) were included in the discrepancy sleep group, and those whose subjective and objective sleep statuses were negative were included in the unfavorable sleep group. Notably, within the discrepancy sleep group, the number of participants with positive subjective but negative objective sleep status was minimal (3.13%). For numeric variables, group differences were assessed using the Kruskal–Wallis test followed by Dunn′s post hoc test. Categorical variables were analyzed using the chi‐squared test with Bonferroni correction. Statistical significance was set at *p* < 0.05 for all analyses. All statistical analyses were performed using SPSS Version 29.0.2.0 (IBM Corp., 2023).

## 3. Results

**Figure 2:**
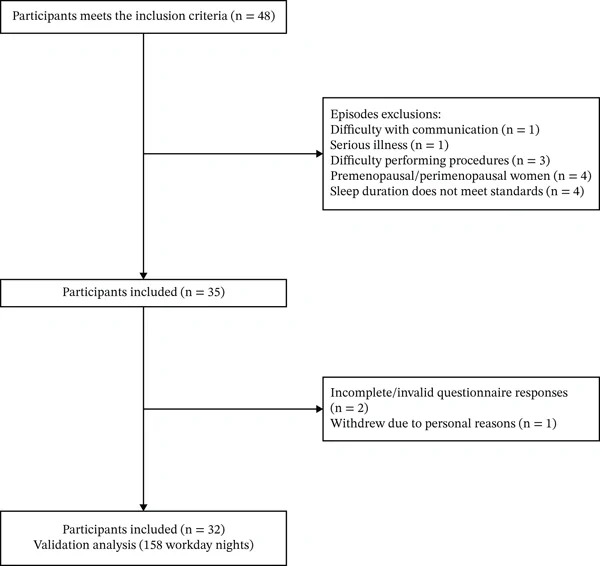
Participant enrollment, exclusion, and analysis flow diagram. Notes: A total of 48 individuals expressed interest in participating in the present study, of whom 13 were excluded based on predefined exclusion criteria. The remaining 35 participants were enrolled and data were collected. During the monitoring period, three participants were excluded from the final analysis—two due to incomplete or invalid questionnaire responses and one due to withdrawal for personal reasons. Ultimately, 32 participants were included in the final analysis, yielding an overall attrition rate of 8.57% (3/35) after enrollment.

Among the 35 participants who met the inclusion criteria, 3 (8.57%) were excluded from the final analysis. Missing data were handled using listwise deletion (Figure 2). A total of 32 participants were included in our final statistical analysis, which compared the male and female groups across several demographic, health, and sleep discrepancies. No significant sex‐related differences were observed in age, BMI, exercise habits, marital status, presence of basic diseases, or perceived health status (Table 1).

**Table 1:** Summary of demographic, sleep discrepancies, and psychological symptoms rating data.

Sleep and sleep‐related features	Total sample (*n* = 32)	Females (*n* = 18)	Males (*n* = 14)	t/Z, *χ* ^2^	*p*
Basic					
Age	55.86 ± 4.98	55.84 ± 4.70	55.88 ± 5.41	−0.02	0.987
BMI	22.37 ± 3.87	21.67 ± 3.52	23.26 ± 4.24	−1.13	0.271
Exercise, mean day/min	38.53 ± 35.54	33.28 ± 30.15	45.00 ± 40.91	−1.95	0.053
Education, *n* (%)	Above high school/high school	5/13 (27.8%)	2/12 (14.3%)	0.27	0.443
Marriage, *n* (%)	Single/living with family	4/14 (22.2%)	5/9 (35.7%)	0.03	0.826
Basic disease, *n* (%)	With/without	7/11 (38.8%)	10/4 (71.4%)	0.07	0.597
SRH, *n* (%)	Positive/negative	8/10 (44.4%)	8/6 (57.1%)	0.03	0.813
Questionnaires					
TMD scores	47.47 ± 4.79	47.00 ± 3.85	48.04 ± 5.77	−0.58	0.571
TA scores	5.00 (3.00,8. 00)	6.00 (2.75, 8.25)	4.00 (3.00, 6.00)	−1.55	0.121
DD scores	2.50 (1.00, 4.00)	3.00 (1.75, 4.00)	2.00 (2.00, 3.75)	−1.43	0.153
AH scores	5.00 (3.00, 6.00)	4.50 (3.00, 6.00)	5.00 (2.25,6.00)	−0.29	0.675
VA scores	8.34 ± 3.73	8.91 ± 3.50	7.65 ± 3.95	1.28	0.206
FI scores	4.00 (3.00, 6.00)	3.00 (3.00, 5.35)	4.00 (2.25, 6.00)	−0.56	0.575
CB scores	5.00 (4.00, 6.00)	5.00 (3.00, 6.00)	5.00 (4.00, 6.75)	−0.70	0.487
Insomnia severity index	6.78 ± 4.12	5.00 (3.75, 7.25)	7.50 (4.00, 11.75)	−1.96	0.050
Sleep discrepancies variable					
SOL discrepancy, mean	6.50 ± 12.17	8.13 ± 9.21	4.41 ± 15.30	0.80	0.436
WASO discrepancy, mean	−10.47 ± 25.7	−6.81 ± 23.61	−14.31 ± 25.80	2.29	**0.010**
TST Misperception Index, mean	0.01 ± 0.09	−0.02 ± 0.09	0.05 ± 0.10	0.94	0.352

*Note:* Baseline characteristics were compared between male and female participants using the *t*‐test or Mann–Whitney *U* test for numeric variables and the chi‐squared test for categorical variables.

Abbreviations: AH, anger–hostility; BMI, body mass index; CB, confusion–bewilderment; DD, depression–dejection; FI, fatigue–inertia; ISI, insomnia severity index; SD, standard deviation; SOL, sleep onset latency; SRH, self‐rated health; TA, tension–anxiety; TMD, total mood disturbance; TST, total sleep time; VA, vigor–activity; WASO, wakefulness after sleep onset.

Statistically significant correlations (*p* < 0.05) are in bold.

Psychological and sleep questionnaire scores, including TMD, TA, DD, AH, VA, FI, CB, and ISI scores, showed no significant sex‐based differences, although ISI scores approached statistical significance (*p* = 0.050), with male participants exhibiting slightly higher scores. In the present study, middle‐aged male participants tended to underestimate WASO time more than female participants (*p* = 0.010).

### 3.1. TST Sleep Misperception Index (Underestimation Vs. Overestimation)

Participants were classified into overestimation and underestimation groups according to their TST sleep misperception index. As shown in Table 2, significant differences were observed in several variables between the two groups. For the demographic factors, participants in the underestimation group were significantly older than those in the overestimation group (Z = −2.14, *p* = 0.032). SRH also differed significantly (*χ* ^2^ = 5.69, *p* = 0.017), with a higher proportion of individuals with self‐reported negative health status in the underestimation group.

**Table 2:** Univariate analyses in the underestimation and overestimation groups.

Variables	Total	Overestimated	Underestimate	t/Z	*p*
Age, M (Q1, Q3)	56.00 (54.00, 60.00)	55.00 (53.25, 57.00)	59.00 (54.75, 61.25)	Z = −2.14	**0.032**
Exercise, M (Q1, Q3)	30.00 (0.00, 60.00)	30.00 (22.50, 60.00)	25.00 (0.00, 30.00)	Z = −1.74	0.082
Sex, *n* (%)	Female/male	9/9 (50.00)	9/5 (64.29)	*χ* ^2^ = 1.38	0.239
BMI, *n* (%)	Normal/abnormal	12/6 (66.67)	5/9 (35.71)	*χ* ^2^ = 3.19	0.074
Marriage, *n* (%)	Single/living with family	6/12 (33.33)	3/11 (21.43)	*χ* ^2^ = 1.84	0.175
Education, *n* (%)	Above high school/high school	3/15 (15.79)	4/10 (28.57)	*χ* ^2^ = 2.32	0.128
Basic disease, *n* (%)	With/without	9/9 (50.00)	8/6 (57.14)	*χ* ^2^ = 0.32	0.571
SRH, *n* (%)	Positive/negative	12/6 (66.67)	4/10 (28.57)	*χ* ^2^ = 5.69	**0.017**
TMD, mean ± SD	47.47 ± 4.79	45.97 ± 4.29	50.10 ± 4.56	*t* = −3.44	**0.001**
TA, M (Q1, Q3)	5.00 (3.00, 8.00)	3.00 (2.00, 6.00)	8.00 (6.00, 9.00)	Z = −4.02	**< 0.001**
DD, M (Q1, Q3)	2.50 (1.00, 4.00)	2.00 (1.00, 4.00)	3.00 (2.00, 4.00)	Z = −1.79	0.073
AH, M (Q1, Q3)	5.00 (3.00, 6.00)	5.00 (3.00, 5.00)	4.00 (2.00, 6.00)	Z = −0.69	0.492
VA, M (Q1, Q3)	8.00 (6.00, 11.00)	10.00 (8.00, 12.00)	7.00 (5.00, 8.00)	Z = −3.18	**0.001**
FI, M (Q1, Q3)	4.00 (3.00, 6.00)	3.00 (2.00, 5.00)	5.00 (3.00, 7.00)	Z = −2.44	**0.015**
CB, M (Q1, Q3)	5.00 (4.00, 6.00)	5.00 (4.00, 6.00)	5.00 (4.00, 7.00)	Z = −0.62	0.534
ISI, M (Q1, Q3)	6.00 (4.00, 9.75)	5.00 (3.00, 7.00)	8.00 (6.00, 12.00)	Z = −2.59	**0.010**

*Note*: Participants were classified into “overestimated” and “underestimated” groups with TST misperception index. The Mann–Whitney *U* test was used for numeric variables and the chi‐squared test for categorical variables.

Abbreviations: AH, anger–hostility; BMI, body mass index; CB, confusion–bewilderment; DD, depression–dejection; FI, fatigue–inertia; ISI, insomnia severity index; SRH, self‐rated health; TA, tension–anxiety; TMD, total mood disturbance; VA, vigor–activity.

Statistically significant correlations (*p* < 0.05) are in bold.

**Figure 3:**
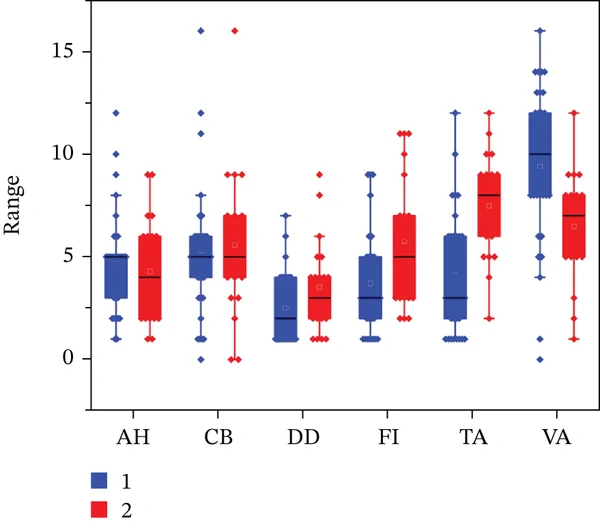
Box line plot of the difference between the overestimated and underestimated sleep groups on the POMS dimension.

Psychological variables showed marked differences: the underestimated group exhibited significantly higher TMD (*t* = −3.44, *p* = 0.001), TA (Z = −4.02, *p* < 0.001), and ISI scores (Z = −2.59, *p* = 0.010). Additionally, they had significantly lower VA scores (Z = −3.18, *p* = 0.001) and higher FI scores (Z = −2.44, *p* = 0.015). No significant differences were found in sex, BMI, marital status, education level, presence of chronic disease, or other POMS subscales, such as DD, AH, and CB scores. Differences in various mood state dimensions between the overestimated and underestimated groups are shown in Figure 3.

### 3.2. Correlation Analysis

Spearman′s correlation analysis revealed significant negative correlations between sleep state misperception index and age and TA, DD, and TMD scores (Table 3). Specifically, weak negative correlations were found between sleep state misperception index and both age and TA and TMD scores (*r* = −0.364,*p* < 0.01; *r* = −0.361, *p* < 0.01; *r* = −0.287, *p* < 0.01), whereas a moderate negative correlation was observed between sleep misperception index and DD scores (*r* = −0.475, p < 0.01). In addition, no significant correlations were found between sleep misperception index and AH, VA, CB, or ISI scores.

**Table 3:** Results of correlation coefficients between psychological/behavioral variables and TST misperception index.

Variables	Age	TA	DD	AH	VA	FI	CB	TMD	ISI	TST misperception index
1. Age	1									
2. TA	0.416 ^∗∗^	1								
3. DD	0.331	0.293 ^∗^	1							
4. AH	− 0.020	0.035	0.179	1						
5. VA	− 0.182	− 0.210	− 0.345 ^∗^	− 0.035	1					
6. FI	0.109	0.417 ^∗∗^	0.340 ^∗^	0.411 ^∗^	− 0.346 ^∗^	1				
7. CB	0.110	0.234	0.130	0.301 ^∗^	− 0.130	0.224	1			
8. TMD	0.362 ^∗^	0.523 ^∗∗^	0.473 ^∗∗^	0.452 ^∗∗^	− 0.497 ^∗∗^	0.501 ^∗∗^	0.543 ^∗∗^	1		
9. ISI	− 0.314 ^∗∗^	0.492 ^∗∗^	0.253	0.317 ^∗^	− 0.370 ^∗^	0.523 ^∗∗^	0.182	0.592 ^∗∗^	1	
10. TST Misperception Index	− 0.364 ^∗∗^	− 0.361 ^∗∗^	− 0.475 ^∗∗^	0.069	0.179	− 0.133	0.112	− 0.287 ^∗∗^	− 0.212	1

Abbreviations: AH, anger–hostility; CB, confusion–bewilderment; DD, depression–dejection; FI, fatigue–inertia; ISI, insomnia severity index; TA, tension–anxiety; TMD, total mood disturbance; TST, total sleep time; VA, vigor–activity.

^∗^Presents *p* < 0.05;

^∗∗^presents *p* < 0.01.

### 3.3. Multiple Regression Analysis for Sleep State Misperception Index

Table 4 summarizes the results of the linear regression analysis conducted to identify the predictors of misperception index. Sex was a predictor of sleep misperception index. Compared with female participants (reference group), male participants had significantly positive sleep discrepancy scores (sleep misperception index > 0), indicating a greater tendency to overestimate sleep duration. In the model, *β* coefficient was 4.25 (standard error [*S* *E*] = 2.03, *t* = 2.13, *p* = 0.042, 95% confidence interval [CI] [0.17, 8.22]). SRH was negatively associated with sleep discrepancies. Participants who perceived themselves to be in negative health states had negative sleep discrepancy scores compared with those who perceived themselves to be in positive health states (reference group), suggesting a tendency to underestimate their sleep duration. In the model, *β* coefficient was −6.10 (*S* *E* = 2.70, *t* = −2.26, *p* = 0.031, 95% CI [−11.67, −0.57]). TMD scores were also significantly associated with negative sleep discrepancy scores, indicating a tendency toward sleep underestimation among individuals with high scores. In the model, *β* coefficient was −1.65 (*S* *E* = 0.58, *t* = −2.86, *p* < 0.008, 95% CI [−2.84, −0.46]). The overall model was statistically significant (*F* = 7.873, *p* = 0.001), with all tolerance values exceeding 0.10 (minimum = 0.709), indicating that multicollinearity among predictors was not a concern in this analysis.

**Table 4:** Multiple linear regression analysis of predictors of TST misperception index.

Variables	*β*	S. E	*t*	*p*	*β*(95% CI)
Sex					
Female					0.00 (reference)
Male	4.25	2.03	2.13	**0.042**	4.25 (0.17, 8.22)
SRH					
Positive					0.00 (reference)
Negative	− 6.10	2.70	− 2.26	**0.031**	− 6.10 (− 11.67, − 0.57)
Total mood disturbance scores	− 1.65	0.58	− 2.86	**0.008**	− 1.65 (− 2.84, − 0.46)

*Note:* Multiple linear regression including all related variables and adjusted for age, BMI, and subjective wakefulness after sleep onset. In the multiple models, *R* ^2^ = 0.458, adjusted *R* ^2^ = 0.392, F = 7.873, *p* = 0.001. All tolerance values were greater than 0.1 (minimum = 0.709), indicating that multicollinearity among the independent variables was not severe. All three variables exhibited moderate effects (Sex: *f* ^2^ = 0.16; SRH: *f* ^2^ = 0.18; TMD: *f* ^2^ = 0.28).

Abbreviations: BMI, body mass index; CI, confidence interval; SRH, self‐rated health; TMD, total mood disturbance; TST, total sleep time.

Statistically significant correlations (*p* < 0.05) are in bold.

### 3.4. Univariate Analysis Based on Presence of Subjective and/or Objective Insomnia

Table 5 shows the results of univariate analysis comparing the favorable sleep, discrepancy sleep, and unfavorable sleep groups. Significant group differences were found for age (*p* = 0.003), with the “ unfavorable sleep” group being the oldest (58.26 ± 4.48 years), followed by the discrepancy group (56.37 ± 4.76 years) and the favorable sleep group (53.5 ± 4.47 years). Post hoc comparisons revealed significant differences between the favorable sleep and unfavorable sleep groups (*p* = 0.007) and between the favorable sleep and discrepancy sleep groups (*p* = 0.036) but not between the discrepancy sleep and unfavorable sleep groups (*p* = 0.997). SRH differed significantly across the groups (*P* = 0.026), with a higher proportion of participants reporting good health in the favorable sleep group (63.6%) than in the discrepancy sleep (40.0%) or unfavorable sleep groups (45.4%). However, post hoc comparisons did not reach significance after Bonferroni correction (*p* = 0.783, *p* = 0.074, *p* = 0.051, respectively).

**Table 5:** Univariate analysis in groups with or without subjective/objective insomnia.

Characteristic	Favorable sleep	Discrepancy sleep	Unfavorable sleep	Kruskal–Wallis/ANOVA	*P*_1_	*P*_2_	*P*_3_
Age (years)	53.5 ± 4.4	56.3 ± 4.7	58.2 ± 4.4	**0.003**	**0.007**	**0.036**	0.997
Sex, female (*n*%)	6/5 (54.6)	5/5 (50.0)	7/4 (63.6)	0.375	0.490	0.985	0.405
Perceived health, health *n* (%)	7/4 (63.6)	4/6 (40.0)	5/6 (45.5)	**0.026**	0.074	0.051	0.983
Questionnaire							
TMD scores	40 (42, 47)	50 (46, 55)	48 (46, 51)	**< 0.001**	**0.049**	0.237	0.764
TA scores	2.5 (1, 4)	**8 (6, 10)**	6 (3, 8)	**< 0.001**	**< 0.001**	**0.007**	**0.047**
DD scores	2 (1, 2.75)	3 (1, 4)	3 (2, 5)	**0.031**	**0.007**	0.229	0.774
AH scores	4(2, 5.75)	4 (2, 7)	5 (3, 6)	0.515	0.855	0.934	0.967
VA scores	9.5 (8, 12.75)	8 (5, 11)	8 (6, 11)	0.297	0.444	0.444	0.973
FI scores	3 (1.25, 3.75)	**5 (3, 9)**	4 (3, 6.75)	**0.004**	**0.020**	**0.049**	0.961
CB scores	4.5 (3, 5.75)	5 (5, 6)	6 (4, 9)	0.129	0.772	0.229	0.916

*Note*: The Kruskal–Wallis test with Dunn′s test was used for numeric variables, and the chi‐squared test with Bonferroni correction was used for categorical variables. *P*_1:_ favorable sleep versus unfavorable sleep; *P*_2_: favorable sleep versus discrepancy sleep; and *P*_3_: discrepancy sleep versus unfavorable sleep. The sleep data in this study were collected over a 2‐week period and should not be interpreted as indicative of a clinical diagnosis of insomnia. The findings reflect sleep tendencies observed during that specific timeframe.

Abbreviations: AH, anger–hostility; CB, confusion–bewilderment; DD, depression–dejection; FI, fatigue–inertia; TA, tension–anxiety; TMD, total mood disturbance; VA, vigor–activity.

Statistically significant correlations (*p* < 0.05) are in bold.

Regarding psychological measures, significant group differences were found in TMD scores (*p* < 0.001), with higher scores in the unfavorable sleep group than in the favorable sleep group. Among the different mood state categories, TA scores (*p* < 0.001) were the highest in the discrepancy sleep group, DD scores (*p* = 0.031) were the highest in the unfavorable sleep group, and FI scores (*p* = 0.004) were the highest in the discrepancy sleep group. Post hoc tests indicated that the favorable sleep group had significantly lower TMD (*p* = 0.049), TA (*p* < 0.001), DD (*p* = 0.007), and FI scores (*p* = 0.020) than the unfavorable sleep group. TA and FI scores also differed significantly between the favorable sleep and discrepancy sleep groups (*p* = 0.007 and *p* = 0.049, respectively). Notably, TA scores were higher in the discrepancy sleep group than in the unfavorable sleep group (*p* = 0.047). No significant differences were found in the AH, VA, or CB scores between the groups.

## 4. Discussion

This study examined the associations between sleep state misperception, emotional states, and health status in community‐dwelling middle‐aged adults, a population in which these associations have not yet been fully evaluated. In this cross‐sectional study involving 32 middle‐aged adults, those who underestimated their TST were older, reported poorer SRH, and exhibited greater fatigue and anxiety compared with those who overestimated their TST. Sex, SRH, and TMD scores emerged as independent predictors of the sleep misperception index. Furthermore, participants in the discrepancy sleep group reported the highest TA and FI scores, suggesting that the subjective–objective sleep discrepancy is more closely associated with specific emotional states than with objective sleep parameters alone.

Previous studies have shown that sleep state misperception is related to emotional arousal among specific groups. Individuals with insomnia tend to underestimate sleep duration, and this underestimation has been linked to heightened emotional arousal and perceptual bias, with a median discrepancy of 46 min (*p* < 0.001) [39]. Subjective sleep indicators have also been reported to be more strongly associated with affective states than objective sleep indicators (*β* [95% CI] = 0.34 [0.11, 0.57]) [40]. The present study extends this evidence to a community‐based middle‐aged sample, demonstrating that sleep underestimation rather than overestimation is more closely associated with recent emotional arousal and negative emotional states. Across mood dimensions, higher TA, DD, and TMD scores demonstrated negative correlation with the misperception index, indicating a tendency toward underestimation of TST (*p* < 0.05), whereas AH, VA, FI, and CB scores showed positive correlations, suggesting a tendency toward overestimation, though these associations did not reach statistical significance. Notably, although TA and depressive affect represent distinct emotional categories in terms of arousal level and valence, both were associated with underestimation of sleep duration, suggesting that mechanistically distinct pathways may converge on the same direction of sleep misperception. Furthermore, TMD scores emerged as an independent predictor of the sleep misperception index, suggesting that the cumulative burden of overall negative mood disturbance, together with specific emotional dimensions, shapes subjective sleep perception in middle‐aged adults. These findings underscore the complexity of the subjective–objective sleep discrepancy and support the utility of sleep misperception measures as accessible indicators of emotional vulnerability in community‐dwelling middle‐aged adults.

Among sleep state misperception‐related factors, sleep underestimation in middle‐aged adults appears to be shaped by the interplay of emotional arousal, SRH, and sex. Emotional arousal alters time perception and amplifies attentional bias toward nocturnal awakenings, biasing subjective sleep estimates downward [41–43]. Poor SRH similarly redirects attention toward bodily sensations during sleep, increasing perceived fragmentation [40, 44], whereas fatigue amplifies awareness of brief awakenings, making sleep feel shorter than it objectively is [45]. These factors may be mutually reinforcing in middle‐aged adults, as declining health perception, emotional distress, and fatigue commonly co‐occur during this life stage. Regarding sex‐based differences, middle‐aged male participants tended to overestimate sleep duration relative to female participants. This pattern likely reflects the convergence of biological, interoceptive, and sociocultural factors. Postmenopausal women experience hormonal shifts, including declining estrogen, progesterone, and testosterone levels, that alter sleep architecture and increase nocturnal awakenings [46], which may heighten their sensitivity to sleep disruption and contribute to a tendency toward underestimation of sleep duration relative to objective measurements. In addition, women have been shown to demonstrate greater interoceptive sensibility than men [47], further facilitating more accurate or negatively biased perception of internal bodily states including sleep. Beyond physiology, men and women may differ in how they conceptualize and report sleep quality, with men potentially more inclined to evaluate sleep in terms of duration and efficiency, whereas women may place greater emphasis on nocturnal disturbances and daytime consequences [48], which could further contribute to sex‐based differences in subjective sleep reporting. Sociocultural norms valorizing stoicism in men may further discourage acknowledgment of sleep difficulties, contributing to systematic overestimation of sleep quality relative to objective measurements [49, 50]. Although anxiety and depression are more prevalent in women and are associated with greater subjective sleep complaints [51], our findings revealed that men reported better subjective sleep than was indicated by objective measurements, suggesting that sleep overestimation in men may obscure genuine sleep‐ and health‐related risks in this population.

Among the subgroups, participants in the discrepancy sleep group reported the highest scores on the TA and FI subdomains of the POMS, compared with those with relatively consistent subjective and objective sleep perceptions. These findings suggest that the subjective–objective sleep discrepancy is more closely associated with specific emotional states than with objective sleep parameters alone. At the cognitive level, negative emotions accompanied by rumination and heightened self‐focused attention may amplify the perceived severity of sleep‐related experiences relative to objective measurements [52, 53]. At the physiological level, anxiety and fatigue have been associated with sympathetic nervous system activation and hypothalamic–pituitary–adrenal axis dysregulation, which may reduce deep sleep proportion and overall sleep efficiency, thereby disrupting the subjective sense of sleep restoration and contributing to greater subjective–objective discrepancy. Notably, the POMS fatigue subscale primarily reflects subjective feelings of tiredness within an emotional context and may not fully capture other dimensions of fatigue, such as physiological daytime sleepiness or functional fatigue severity. Future studies incorporating more specific fatigue instruments, such as the Fatigue Severity Scale or the Epworth Sleepiness Scale, would help clarify the extent to which different fatigue dimensions contribute to sleep misperception and further delineate the boundary conditions under which emotional fatigue influences subjective–objective sleep discrepancy in middle‐aged community populations.

Based on these associations, the sleep state misperception index, derivable from consumer‐grade wearable devices and sleep diaries, may represent a practical and scalable approach for population‐level emotional health screening in community settings, particularly within aging societies where accessible monitoring tools are increasingly needed. The observed sex‐related differences further suggest that screening strategies may need to be sex‐specific, as sleep overestimation among men may systematically obscure underlying emotional and health‐related risks. For individuals presenting with sleep misperception alongside negative emotional states, interventions targeting both perceptual biases and emotional dysregulation may support broader emotional well‐being beyond sleep improvement alone. Cognitive behavioral therapy for insomnia has demonstrated effectiveness in reducing not only insomnia severity but also negative affect and emotional regulation difficulties, whereas complementary approaches including Mindfulness‐Based Stress Reduction, Acceptance and Commitment Therapy, and biofeedback may further address arousal‐based mechanisms underlying sleep misperception, with digital mindfulness interventions showing particular promise as scalable tools for community‐level implementation. Nevertheless, the utility of sleep state misperception as a screening indicator warrants validation in larger community samples, and longitudinal designs are needed to establish the temporal directionality of these associations before clinical recommendations can be established.

This study has some limitations. First, the relatively limited sample size warrants cautious interpretation of the findings, and studies with larger cohorts will be necessary to verify these findings. Second, the cross‐sectional design precludes causal inference regarding the directionality of associations between sleep misperception and emotional states, and the temporal dynamics of these relationships could not be examined. Third, although the Polar Vantage was selected for its validated performance in community‐based settings, its accelerometry‐based algorithms may misclassify quiet wakefulness as light sleep and show limited accuracy in sleep stage classification, particularly for rapid eye movement sleep. Fourth, the exclusion of sleep‐related breathing disorders was based on previous clinical PSG findings, rather than prospective standardized assessment, and baseline respiratory disturbance index data were unavailable. Consequently, subtle sleep‐related breathing disorders cannot be completely excluded as a potential contributor to subjective sleep complaints. Fifth, psychiatric disorders were excluded based on self‐report, rather than a structured diagnostic interview. Therefore, undiagnosed psychiatric conditions cannot be completely excluded, and future studies should consider using standardized instruments such as the Mini‐International Neuropsychiatric Interview. Sixth, although specific inclusion criteria were applied when recruiting middle‐aged women, controlling for all potential hormonal confounders that may influence sleep and emotional states remained impossible.

Future research should build upon the associations identified in the present study by addressing the methodological limitations. First, longitudinal designs would help clarify the temporal directionality and potential causal relationships between sleep misperception and emotional states. Second, future research should be directed toward establishing a standardized framework for quantifying the subjective–objective sleep discrepancy using consumer‐grade sleep tracking devices. Specifically, it would be valuable to determine which objective sleep parameters most effectively capture variations in sleep perception patterns, identify appropriate methods for their quantification, and define clinically meaningful threshold values. Such efforts would enable reliable sleep monitoring in community settings and facilitate the differentiation between underestimation, overestimation, and normative perception groups. This approach may support the development of CST‐based digital health platforms that leverage low‐burden devices to enable large‐scale, continuous, community‐based longitudinal monitoring, allowing integrated collection and analysis of sleep, physiological, and behavioral data to support long‐term health management in community populations. Achieving this goal will require rigorously designed experimental paradigms to standardize measurement procedures and reduce error, alongside longitudinal designs to characterize the temporal structure and potential gradient nature of sleep misperception in relation to emotional states.

## 5. Conclusions

In summary, our study identified moderate associations between emotional states, including anxiety and fatigue, and sleep misperception. Among community‐dwelling middle‐aged adults, poorer SRH and greater emotional fluctuation were linked to a higher likelihood of underestimating sleep duration, with sex acting as an additional contributing factor. Individuals showing subjective–objective sleep discrepancies also exhibited greater variability in anxiety and fatigue, highlighting the need for further causal investigation. In community sleep health management, sleep misperception indicators show an association with emotional vulnerability. Long‐term, low‐burden, high‐durability actigraphy monitoring could support earlier identification to improve sleep health in middle‐aged populations in the future.

## Author Contributions

Xiaoyu Sun: Conceptualization, methodology, data curation, writing—original draft preparation. Hideki Miyaguchi: Conceptualization, supervision. chinami ishizuki: supervision. Hitoshi Okamura: Conceptualization, methodology, writing—reviewing and editing. All authors contributed to manuscript revision.

## Funding

No funding was received for this manuscript.

## Disclosure

All authors read the manuscript and approved the submitted version.

## Ethics Statement

All procedures involving human participants were conducted in accordance with the ethical standards of the institutional research committee and with the 1964 Helsinki Declaration and its later amendments. This study was reviewed and approved by the Ethics Committee of Hiroshima University (Approval No. E2024‐0193).

## Consent

Written informed consent was obtained from all participants prior to their inclusion in the study.

## Conflicts of Interest

The authors declare no conflicts of interest.

## Data Availability

The data that support the findings of this study are available on request from the corresponding author. The data are not publicly available due to privacy or ethical restrictions.
